# Particulate Air pollution mediated effects on insulin resistance in mice are independent of CCR2

**DOI:** 10.1186/s12989-017-0187-3

**Published:** 2017-03-03

**Authors:** Cuiqing Liu, Xiaohua Xu, Yuntao Bai, Jixin Zhong, Aixia Wang, Lixian Sun, Liya Kong, Zhekang Ying, Qinghua Sun, Sanjay Rajagopalan

**Affiliations:** 1 Basic Medical College, Zhejiang Chinese Medical University, 548 Binwen Rd, Building 15#, Room 303, Hangzhou, 310053 China; 2 Wexner Medical Center, The Ohio State University, Columbus, OH USA; 3 Division of Cardiovascular Medicine, University of Maryland, Baltimore, MD USA; 4 Division of Cardiovascular Medicine, The Affiliated Hospital of Chengde Medical College, Chengde, China; 5 College of Public Health, Division of Environmental Health Sciences, The Ohio State University, Columbus, OH USA; 6 Cardiovascular Research Institute, Case Western Reserve School of Medicine, 11100 Euclid Avenue, Cleveland, OH 44106 USA

**Keywords:** Particulate matter, Insulin resistance, CCR2, Normal diet, Inflammation, Gluconeogenesis

## Abstract

**Background:**

Chronic exposure to fine ambient particulate matter (PM_2.5_) induces insulin resistance. CC-chemokine receptor 2 (CCR2) appears to be essential in diet-induced insulin resistance implicating an important role for systemic cellular inflammation in the process. We have previously suggested that CCR2 is important in PM_2.5_ exposure-mediated inflammation leading to insulin resistance under high fat diet situation. The present study assessed the importance of CCR2 in PM_2.5_ exposure-induced insulin resistance in the context of normal diet.

**Methods and Results:**

C57BL/6 and CCR2^-/-^ mice were subjected to exposure to concentrated ambient PM_2.5_ or filtered air for 6 months. In C57BL/6 mice, concentrated ambient PM_2.5_ exposure induced whole-body insulin resistance, macrophage infiltration into the adipose tissue, and upregulation of phosphoenolpyruvate carboxykinase (PEPCK) in the liver. While CCR2 deficiency reduced adipose macrophage content in the PM_2.5_-exposed animals, it did not improve systemic insulin resistance. This lack of improvement in insulin resistance was paralleled by increased hepatic expression of genes in PEPCK and inflammation.

**Conclusion:**

CCR2 deletion failed to attenuate PM_2.5_ exposure-induced insulin resistance in mice fed on normal diet. The present study indicates that PM_2.5_ may dysregulate glucose metabolism directly without exerting proinflammatory effects.

**Electronic supplementary material:**

The online version of this article (doi:10.1186/s12989-017-0187-3) contains supplementary material, which is available to authorized users.

## Background

Accumulating evidence implicates adverse cardiometabolic consequences of fine particulate matter (PM_2.5_) exposure, including worsening of insulin sensitivity and/or visceral inflammation/adiposity in mice irrespective of diets [1–4]. It is well known that insulin resistance (IR) is often coupled to a cellular inflammatory response in insulin sensitive tissues including liver and adipose tissue, driving the development of abnormalities in glucose and lipoprotein metabolism [5–8].

Monocyte chemoattractant proteins (MCPs) and their receptors play a pivotal role in tissue inflammatory responses via recruitment of monocytes to sites of inflammation [9]. Recent studies have demonstrated that C-C Motif Chemokine Ligand 2 (CCL2, also known as MCP1) inhibits insulin-stimulated glucose uptake as well as the adipocyte expression of genes implicated in tissue IR and dysregulated metabolism [10]. In high fat diet (HFD) fed mice, genetic deficiency in CC-chemokine receptor 2 (CCR2, the receptor of MCP1) reduces macrophage content and inflammation while simultaneously improving glucose homeostasis and insulin sensitivity suggesting that cellular inflammation mediated via CCR2 is coupled to development of IR and metabolic dysregulation [7]. In these studies, long periods of HFD feeding is typically required before CCR2 deficiency-mediated differences in macrophage recruitment and improvements in insulin sensitivity are observed, despite their striking reduction in circulating inflammatory monocytes.

Consistent with this concept, our previous study demonstrated a protective role of CCR2 deficiency in mice fed HFD and exposed to PM_2.5_ inhalation over a duration of 17 weeks [3]. CCR2^-/-^ mice have a defect in the egress of inflammatory monocytes from the bone marrow, resulting in a dramatic decreased pool of circulating inflammatory monocytes and reduced numbers of differentiated myeloid cells recruited to sites of inflammation which may modulate the impact of inflammatory insults including diet and other environmental factors. Given the importance of HFD in promoting cellular inflammation in insulin responsive tissues particularly over prolonged periods, we were interested in further understanding the influence of CCR2 in the context of normal diet (ND) over long periods of air pollution exposure to address the role of CCR2 in air pollution mediated alterations in IR and cellular inflammation. Accordingly, as a continuation of our previous work with HFD fed mice [3], we examined the effects of CCR2 deficiency on development of PM_2.5_ exposure-induced IR in wild-type and CCR2^-/-^ mice fed ND.

## Results

### PM_2.5_ exposure characterization

**Fig. 1:**
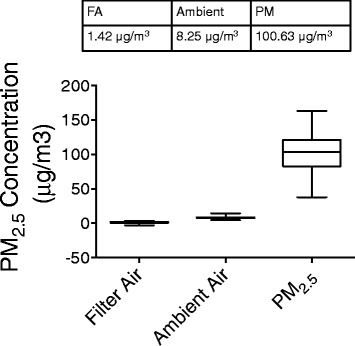
PM_2.5_ concentration to which mice were exposed at the study site

To assess the role of CCR2 in the development of IR, CCR2^-/-^ mice were exposed to PM_2.5_ in parallel with C57BL/6 mice. Ambient mean daily PM_2.5_ concentration at the study site was 8.25 ± 2.12 μg/m^3^. The mean concentration of PM_2.5_ in the filter air chamber was 1.42 ± 1.44 μg/m^3^. The mean concentration of PM_2.5_ in the exposure chamber was 100.63 ± 30.81 μg/m^3^ (12.2-fold concentration from ambient PM_2.5_ level) (Fig.1). Detailed elemental characterization of the exposure environment during the exposure period can be found in Additional file 1: Table S1.

### Effect of PM_2.5_ exposure on glucose metabolism and insulin sensitivity in C57BL/6 and CCR2^-/-^ mice

**Fig. 2:**
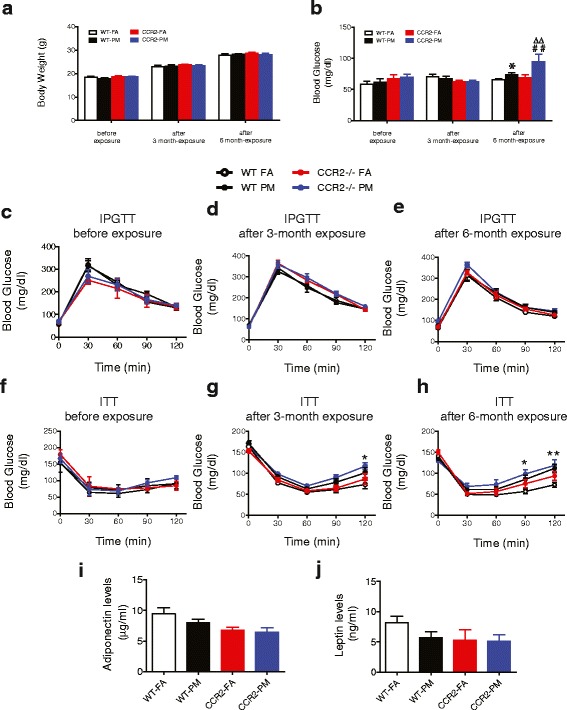
Effect of PM_2.5_ exposure on glucose homeostasis in WT and CCR2^-/-^ mice. **a**, Body weight of mice at baseline, at the end of 3 and 6-months exposure to PM_2.5_ or FA. **b**, Fasting blood glucose at baseline, at the end of 3 and 6-months exposure to PM_2.5_ or FA. **c**-**e**, Intraperitoneal glucose tolerance test (IPGTT) in overnight fasted mice (**c**) before, (**d**) at the end of 3-months, and (**e**) at the end of 6-months PM_2.5_ exposure. **f**-**h**, Insulin tolerance test (ITT) in 4.5 h fasted mice (**f**) before, (**g**) at the end of 3-months, and (**h**) at the end of 6-months PM_2.5_ exposure. **i**-**j**, Plasma adiponectin (**i**) and leptin (**j**) levels at the end of PM_2.5_ exposure. *P < 0.05, ** *P* < 0.01, *** *P* < 0.001 when WT-PM compared to WT-FA group; ## *P* < 0.01 when compared CCR2-PM to WT-PM group; ∆∆ *P* < 0.01 when compared CCR2-PM to CCR2-FA group. *n* = 5–10 per group

There was no significant difference among the four groups in body weight, fasting blood glucose, glucose tolerance or insulin sensitivity at baseline prior to assignment to exposure protocols (Fig. 2a, b, c and f). PM_2.5_ inhalation over 24 weeks resulted in no difference in body weight in both WT and CCR2^-/-^ animals (Fig. 2a). Alterations in fasting blood glucose were not noted until 6 months in response to PM_2.5_ exposure in WT animals (*P* < 0.05, Fig. 2b) but without alterations in glucose tolerance at both 3 and 6 months (Fig. 2d and e). Interestingly, after 6-months PM_2.5_ exposure, blood glucose was significantly elevated in mice from CCR2-PM group and higher than that in WT-PM group. In contrast, CCR2^-/-^ did not alter fasting glucose at either the 3 or 6 months’ time points in the absence of PM_2.5_ exposure (Fig. 2b).

Compared with WT-FA group, animals from WT-PM resulted in abnormal whole body insulin sensitivity after 3-months PM_2.5_ exposure, as evidenced by higher glucose levels 120 min after insulin injection (*P* < 0.05, Fig. 2g). Longer durations of exposure over 6-months impaired insulin sensitivity to a greater extent (*P* < 0.01) and at earlier time points ([90 min (*P* < 0.05) after insulin injection] (Fig. 2h). Insulin tolerance testing in CCR2-PM group revealed significantly higher glucoses at both 90 min and 120 min even at the end of 3-months PM_2.5_ exposure (*P* < 0.05, *P* < 0.01 respectively, Fig. 2g) and continued to be at ahigh level in CCR2-PM group after 6-months PM_2.5_ exposure (Fig. 2h), indicating CCR2 deletion did not improve PM_2.5_-impaired insulin sensitivity over longer durations. Although no significant difference was observed in WT-PM group compared to that in WT-FA group, levels of plasma adiponectin or leptin showed a clear trend toward decrease in CCR2 knockout mice (Fig. 2i and j).

### Effect of PM_2.5_ exposure on systemic inflammation in C57BL/6 and CCR2^-/-^ mice

**Fig. 3:**
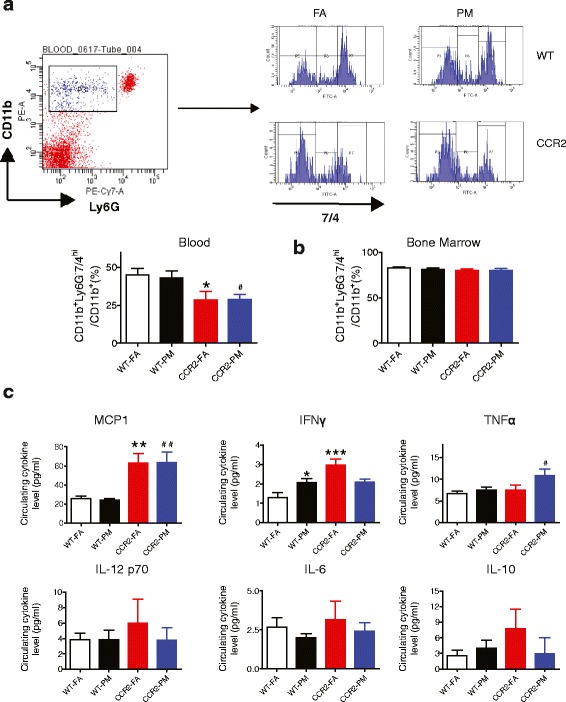
Effects of PM_2.5_ exposure on inflammation in blood and bone marrow from WT and CCR2^-/-^ mice. **a**, Representative flow-cytometric dot plots and analysis showing circulating CD11b^+^Ly6G^−^7/4^hi^ cells from mice blood at the end of 6-months PM_2.5_ exposure. **b**, Analysis showing CD11b^+^Ly6G^−^7/4^hi^ cells from mice bone marrow at the end of 6-months PM_2.5_ exposure. **c**, The concentrations of circulating inflammatory cytokines (MCP-1, IFNγ, TNFα, IL-12 p70, IL-6, IL-10) at the end of PM_2.5_ exposure. *P < 0.05, **P < 0.01, ***P < 0.001 compared to WT-FA group, #*P* < 0.05, ##*P* < 0.01 when CCR2-PM compared to WT-PM group. *n* = 6–9 per group

We examined the effects of PM_2.5_ exposure on inflammatory monocytes in bone marrow and blood. In the present study, we defined monocytes as side scatter-low, forward scatter-high cells expressing the myeloid antigen 7/4 (high populations) and high levels of CD11b but showing no expression for the neutrophil marker Ly6G. The CD11b^+^Ly6G^−^7/4^hi^ cells correspond to Ly6C^hi^ monocytes, representing the inflammatory subtype [11]. Although no difference was observed in WT-PM compared with WT-FA, our results showed a marked decrease in this monocyte subtype in blood from CCR2^-/-^ animals, in both FA and PM groups (*P* < 0.05, Fig. 3a). However, there was no difference of this subset in bone marrow (Fig. 3b) in response to PM_2.5_ exposure in either WT or CCR2^-/-^ animals. Next, we measured inflammatory cytokines in the blood to examine systemic cytokine release in response to PM_2.5_ exposure. As shown in Fig. 3c, although there was no difference in plasma level of MCP-1 between WT-FA group and WT-PM, it significantly increased with CCR2 deletion, regardless of PM_2.5_ exposure (*P* < 0.01 for both groups compared to WT-FA, and *P* < 0.01 when CCR2-PM compared with WT-PM). PM_2.5_ exposure induced significant elevation of IFNγ in both WT (*P* < 0.05) and CCR2 deficiency mice (*P* < 0.05). Interestingly, IFNγ showed an elevated level in CCR2-FA mice too (*P* < 0.001, compared to WT-FA), but with no significant difference between CCR2-FA and CCR2-PM groups. Although TNFα in plasma showed no increase in WT-PM group, it was significantly elevated in CCR2-PM group (both *P* < 0.05 when compared to WT-FA group or WT-PM group). No significant difference was observed in the plasma levels of IL-12p70, IL-6 or IL-10.

### Effect of PM_2.5_ exposure on insulin sensitivity and inflammation in viseral adipose tissue from C57BL/6 and CCR2-/- mice

**Fig. 4:**
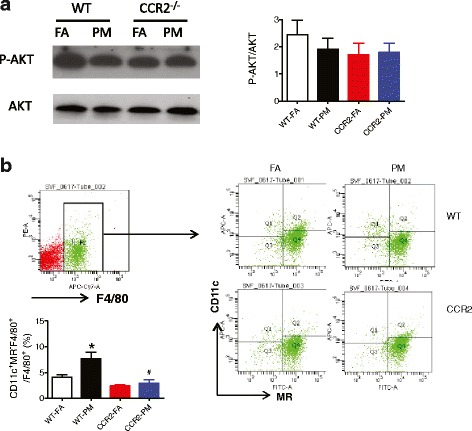
Effect of PM_2.5_ exposure on insulin signaling and inflammation in VAT from WT and CCR2^-/-^ mice. **a**, Representative bands and analysis of western blotting for phosphorylated AKT (P-AKT)/total AKT. **b**, Representative flow-cytometric dot plots showing F4/80^+^/CD11c^+^/MR^−^ with analysis by relative percentage. *P < 0.05, compared to WT-FA group, #*P* < 0.05 when CCR2-PM compared to WT-PM group. *n* = 4–6 per group

Due to aforementioned observation that PM_2.5_ exposure led to systemic alterations in IR, we further tested pathways that represent insulin sensitivity in adipose. No difference was observed in the phosphorylated level of AKT (marker of insulin sensitivity) between groups (Fig. 4a). Next, we examined the activated macrophages defined as F4/80^+^/CD11c^+^/MR^−^ cells in the visceral adipose tissue (VAT). As shown in Fig. 4b, this population was markedly higher in response to PM_2.5_ exposure (*P* < 0.05), which was abolished by CCR2 deficiency (*P* < 0.05). These results indicated that macrophage accumulation, but not AKT phosphorylation, in adipose tissue is modulated in a CCR2-dependent manner in response to air pollution exposure.

### Effect of PM_2.5_ exposure on IR, oxidative stress and inflammation in the liver from C57BL/6 and CCR2^-/-^ mice

**Fig. 5:**
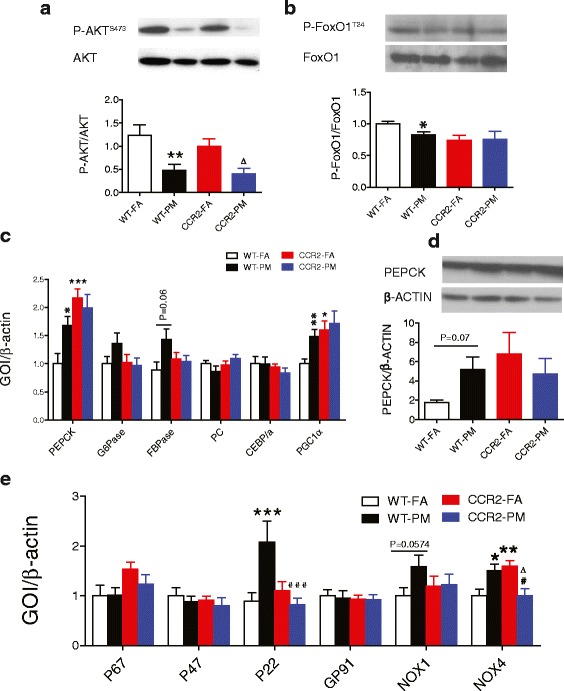
Effect of PM_2.5_ exposure on insulin signaling pathway, gluconeogenesis-related molecules and expression of NADPH oxidase subunits in liver from WT and CCR2^-/-^ mice. **a**-**b**, Representative bands and analysis of western blotting for (**a**) phosphorylated (P-AKT)/total AKT and (**b**) phosphorylated (P-FoxO1)/total FoxO1 in the liver. **c**, mRNA levels of PEPCK, G6Pase, FBPase, PC, CEBPα and PGC1α in the liver of mice. **d**, Protein levels of PEPCK in the liver. **e**. Gene expression of NADPH oxidase subunits in the liver. *P < 0.05, **P < 0.01, ***P < 0.001 compared to WT-FA group; #*P* < 0.05, ###*P* < 0.001 when CCR2-PM compared to WT-PM group; ∆ *P* < 0.05 when compared CCR2-PM to CCR2-FA group. *n* = 5–7 per group for western blotting and *n* = 6–9 per group for gene expression

Significant inhibition in the phosphorylation of Akt in response to PM in both WT and CCR2^-/-^ mice were noted (*P* < 0.01 and *P* < 0.05 respectively, compared to respective FA group Fig. 5a). Given the differences in fasting hyperglycemia without changes in glucose tolerance, we explored gluconeogenesis pathways as important mediators of counter regulatory responses. Figure 5C depicts changes in mRNA expression of key gluconeogenic enzymes and shows significant increase in phosphoenolpyruvate carboxykinase (PEPCK) in response to PM_2.5_ exposure (*P* < 0.05)(Fig. 5c) which was confirmed by measuring its protein level (*P* = 0.07) (Fig. 5d). Further a clear trend toward increase in FBPase in the liver of WT-PM animals (*P* = 0.06), compared to that from WT-FA group, was noted (Fig. 5c). Consistent with it, the transcriptional coactivator PGC1α were markedly increased in liver of WT-PM animals (*P* < 0.01, Fig. 5c). CCR2^-/-^ mice demonstrated higher expression in PEPCK (*P* < 0.001 of CCR2-FA compared with WT-FA; *P* < 0.05 of CCR2-PM compared with WT-FA) and PGC1α (*P* < 0.05 for both groups compared with WT-FA), but no difference in expression of other gluconeogenic genes (G6Pase, FBPase, PC or the transcription factor, C/EBPα, Fig. 5c). The insulin-stimulated phosphorylation of Akt substrates FoxO1 was significantly reduced in WT-PM compared to WT-FA animals (*P* < 0.05). CCR2 deficiency shows no reversion of phosphorylated levels of FoxO1 compared to WT-FA group (Fig. 5b).

PM_2.5_ is known to induce prooxidant and proinflammatory actions, which mediates IR development. To investigate oxidative stress in response to PM_2.5_ exposure, we examined the expression of subunits of NADPH oxidase in the liver. As shown in Fig. 5e, PM_2.5_ exposure induced upregulation of subunit P22 (*P* < 0.001), NOX1 (*P* = 0.0574) and NOX4 (*P* < 0.05), with these changes partially or completely blocked by CCR2 deficiency. Although increased NOX4 level was blocked in CCR2-PM group (*P* < 0.05 when compared with WT-PM), its expression in CCR2-FA group was significantly higher when compared with WT-FA group (*P* < 0.01) or CCR2-PM group (*P* < 0.05). We found no difference in expression of P67, P47 or GP91.

**Fig. 6:**
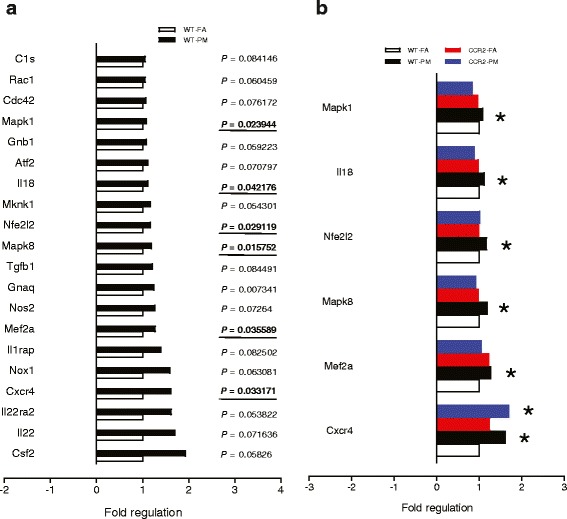
Effect of PM_2.5_ exposure on inflammatory gene expression in liver of WT and CCR2^-/-^ mice. **a**, Top 20 of 179 analyzed genes found increased in response to chronic PM_2.5_ exposure in wild type mice. **b**, Six analyzed genes found to have a significant change from Fig. 6a and the alteration in WT and CCR2^-/-^ mice. *P < 0.05, compared to WT-FA group. *n* = 6 per group

To determine the potential inflammatory basis of PM_2.5_ exposure effects in the liver, we examined a panel of 179 genes involved in inflammation-related mouse genes with a mouse inflammation array. Figure 6a shows the top 20 genes up-regulated by PM_2.5_, only 6 genes of which were increased significantly, including chemokine (C-X-C motif) receptor 4 (Cxcr4), myocyte enhancer factor 2A, transcript variant 4 (Mef2a), mitogen-activated protein kinase 8 (Mapk8), nuclear factor, erythroid derived 2, like 2 (Nfe2l2), interleukin 18 (Il18) and mitogen-activated protein kinase 1 (Mapk1) (Fig. 5a). In the 6 genes, Mef2a, Mapk8, Nfe2l2, Il18 and Mapk1 were normalized or partially normalized in CCR2-/- mice, while Cxcr4 continued to be highly expressed in CCR2-PM group (Fig. 6b).

**Fig. 7:**
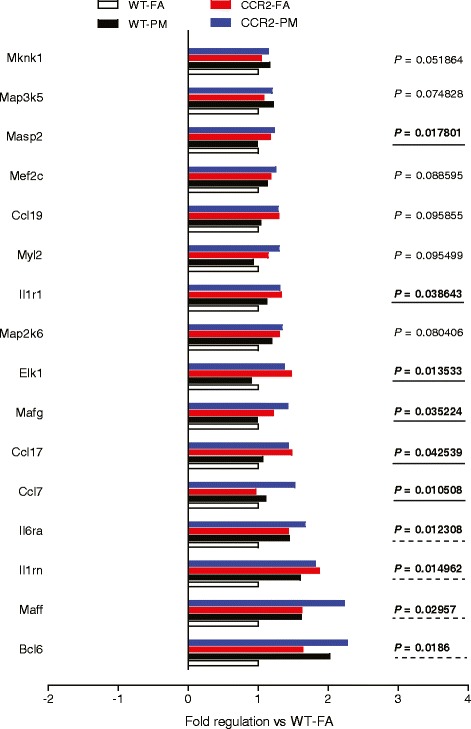
Inflammatory genes with expression increased in CCR2-PM. P values were compared to WT-FA. *n* = 6 per group

To understand in detail, transcriptional responses that may potentially contribute to persistent IR in CCR2 deficiency, we sorted the genes that were up-regulated in the liver of CCR2-PM mice compared to WT-FA group, and analyzed the relationship between this alteration in CCR2-PM and WT-PM groups (with *P* < 0.1). We found expression of some of these genes in CCR2-PM mice were actually increased as WT-PM group did (Fig. 7). These genes include B-cell leukemia/lymphoma 6 (Bcl6), v-maf musculoaponeurotic fibrosarcoma oncogene family, protein F (avian) (Maff), interleukin 1 receptor antagonist (Il1rn), and interleukin 6 receptor, alpha (Il6ra). Although expression of some of other genes demonstrated no increase in WT-PM group compared to WT-FA mice, these genes were upregulated significantly in CCR2 deficiency mice, either PM group only or both FA and PM groups (Fig. 7). These genes include chemokine (C-C motif) ligand 7 (Ccl7), chemokine (C-C motif) ligand 17 (Ccl17), v-maf musculoaponeurotic fibrosarcoma oncogene family, protein G (avian), transcript variant 3 (Mafg), member of ETS oncogene family (Elk1), interleukin 1 receptor, type I (Il1r1), and mannan-binding lectin serine peptidase 2 (Masp2).

## Discussion

In the present study, we note multiple findings that provide an understanding of PM_2.5_ mediated effects on whole body IR in mice. The findings include: (1) Exposure to PM_2.5_ for 6 months induces whole body impairment in insulin responses but not intraperitoneal glucose tolerance; (2) CCR2 deficiency failed to improve PM_2.5_-impaired insulin sensitivity and exaggerated PM_2.5_-induced hyperglycemia in mice. (3) CCR2 deficiency inhibited macrophage infiltration in VAT. (4) However, CCR2 deficiency showed both inhibitory and exaggerated effects on inflammation in the liver, accompanied with dysregulation of gluconeogenesis by targeting the rate limiting enzyme of PEPCK.

The link between exposure to environmental factors in air and propensity to type 2 diabetes has gained only recent attention [1, 2, 12, 13]. To mimic real-world chronic exposures over a good part of rodent lifespan, we exposed 2-months old mice for a 6-months period and investigated its association with IR with the whole body inhalational exposure system. Unlike the impairment in the glucose tolerance after 10 months exposure with adult mice [2] or early-life exposure for 10 weeks with weanling mice [14], PM_2.5_ exposure showed no effect on glucose tolerance in the present study indicating that early-life exposure, exposure duration and diet are major determinants of disturbed glucose metabolism. However, PM_2.5_ inhalation worsened whole-body insulin responsiveness in the present study, providing evidence for an important interaction between environment and metabolism.

In addition to the impaired insulin sensitivity, we demonstrated increased or a trend toward increase in expression of gluconeogenesis-related enzymes including PEPCK and FBPase. To our knowledge, it is the first report that PM_2.5_ exposure leads to enhanced gluconeogenesis in the liver, which is completely different from the study which showed no alteration of PEPCK expression in HFD fed mice exposed to PM_2.5_ [3]. Analyzing the basic expression of PEPCK in mice fed with different diets, about 15-fold elevation of PEPCK expression was shown in HFD situation compared with that in normal chow situation (the relative mRNA expression: 0.59 ± 0.20 in HFD vs 0.04 ± 0.02 in ND) [3]. The different effect of PM_2.5_ on PEPCK expression in the two studies may be related to the fact that the effects of PM_2.5_ on gluconeogenesis may be obscured by a much larger effect of diet. That is, in the presence of HFD feeding, the weak effects of PM_2.5_-increased gluconeogenesis may be overwhelmed. FoxO1 is a transcription factor that functions with the transcriptional cofactor PGC1α to powerfully regulate gluconeogenesis [15, 16]. When FoxO1 is phosphorylated by Akt, it is excluded from the nucleus and gets ubiquitinated and degraded resulting in inhibition of gluconeogenesis and decreased hepatic glucose production [17]. Our data demonstrates abnormal expression of gluconeogenesis-related enzymes, PGC1α and decreased phosphorylated levels of FoxO1 in the liver, suggesting defects in the hepatic insulin-AKT-FoxO1 signaling pathway resulting in enhanced gluconeogenesis and systemic hyperglycemia in response to PM_2.5_ inhalation.

PM_2.5_ is well known to induce prooxidant and proinflammatory actions. Except for the increased macrophage infiltration into the adipose tissue. multiple up-regulated inflammatory genes from gene expression arrays provide more hints into mechanisms by which PM_2.5_ induced IR. Firstly, Nfe2l2 (also known as Nrf2), an antioxidant transcription factor [18], was increased in the liver (Fig. 6), which is consistent with our previous study that long-term PM_2.5_ exposure led to Nrf2-mediated phase II antioxidant responses and may represent a counterregulatory response to circumvent increased oxidant stress [2]. Taken together with the up-regulation of NADPH oxidase subunits (P22, NOX1 and NOX4) (Fig. 5E), it indicates a pathophysiologic role of enhanced oxidant stress in the air pollution-induced IR as demonstrated previously [14, 19, 20]. MAPK1 and MAPK8 (also known as ERK2 and JNK1) are involved in cellular responses to a diverse array of stimuli including proinflammatory cytokines and oxidative stress [21]. Indeed JNKs play a key role in the pathogenesis of hepatic IR and may partly underlie the effects of air pollution. We have previously demonstrated an upregulation of JNK1 in response to air pollution in the liver [19]. The fact that CCR2 deficiency reduces JNK1 expression strongly supports a role for inflammatory infiltration in the liver as a major mechanism of JNK activation. Cxcr4, IL-18 and Mef2 play an important function in the inflammatory responses upon injury or stress by mediating mobilization of multiple cells including progenitor cells into peripheral tissues and serve to amplify cytokine responses such as IFNγ and MCP1 expression, which have been demonstrated to be associated with IR [22–24].

Another important observation in the present study is that PM_2.5_ exposure induced inflammation in the adipose and the liver (MAPK1, MAPK8, IL-18, Nfe2I2 and Mef2a) was abrogated in CCR2 deficiency mice (Fig. 6), while the PM_2.5_ exposure induced IR still ensued. The paradox may be elucidated from the several aspects. (1) The upregulation of a number of inflammatory molecules (CCL7, CCL17, Mafg, Elk1, Masp2, IL1r1, Cxcr4, Bcl6, IL1rn, IL6ra and Maff) in CCR2-PM group. Although not significant, expression of some (Cxcr4, Bcl6, IL1rn, IL6ra and Maff) increased slightly in response to PM_2.5_ exposure in C57BL/6 mice, while expression of the others (CCL7, CCL17, Mafg, Elk1, Masp2 and IL1r1) unaltered. These results may contribute to the hyperglycemia in CCR2-PM animals. (2) The abnormal high level of plasma TNFα in CCR2^-/-^ mice. Innate immunity is regulated in many steps, one of which is the cytokine network with TNFα playing important roles. Whether and how the increased TNFα cytokine leads to some other inflammatory pathways remains unknown. (3) The defects in the hepatic insulin-AKT-FoxO1-gluconeogenesis signaling pathway in CCR2-/- mice. These data demonstrate that PM_2.5_ may exert direct dysregulation of glucose metabolism independent of inflammation. n The alternative receptors or pathways activated by MCP-1. Heesen et al showed that subnanomolar concentrations of MCP-1 specifically induced mouse astrocyte chemotaxis in CCR2-deleted astrocytes [25]. Similarly, Schecter et al reported that MCP-1 stimulated tissue factor mRNA expression and activity in human aortic smooth muscle cells by activating MAPK pathway in the absence of detectable CCR2 mRNA [26, 27]. Given that CCL2 (MCP-1) levels in plasma in CCR2-/- mice were elevated (Fig. 3C), alternative pathways could mediate the effect of MCP-1 in PM_2.5_-induced response. Taken together, it is possible that the hyperglycemia (Fig. 2b) and lack of improvement in IR noted with CCR2^-/-^ may be related to direct enhancement of gluconeogenesis and persistent activation of alternate inflammatory circuits in the liver.

## Limitations and conclusions

Although our results suggest a lack of protective effect of CCR2 deletion in PM_2.5_-mediated IR under ND conditions, several questions remain to be addressed. These include the differential roles of CCR2 under conditions of ND and HFD and precise identification of alternate inflammatory responses that are independent of CCR2. Further delineation of the precise signaling pathways involved in PM_2.5_ mediated inflammatory effects will take additional investigation (MAPK1, MAPK8, IL-18, Nfe2I2, Mef2a). In particular, identifying specific hallmarks of PM_2.5_ mediated impairment in insulin sensitivity that would differentiate from diet-mediated effects may allow design of specific interventions to abrogate PM effects. The relevance of Cxcr4, Bcl6, Maff, IL1rn, IL1r1, IL-6ra, CCL17, Mafg, EIk1 and Masp2) and their exact role in the PM_2.5_–induced IR in absence of CCR2 will also need further exploration. Finally we cannot rule out the effects of strain specific differences in responses that may have contributed to differences noted in this study.

In summary, our results demonstrate that CCR2 deficiency failed to attenuate PM_2.5_ exposure-induced IR in mice. This may be due to the direct dysregulation of gluconeogenesis and non-CCR2 mediated inflammation in the liver. Further exploration of the CCR2-independent inflammatory mechanisms will be informative for determining the targets contributing to impaired metabolism response to PM_2.5_ inhalation.

## Methods

### Animals and animal care

Male C57BL/6 and CCR2-/- mice of 8-week-old (both from Jackson Laboratories, Bar Harbor, Me) were used in this study. All mice were maintained at 21 °C on a 12-h light/12-h dark cycle with free access to water and food. The protocols and the use of animals were approved by and in accordance with the Ohio State University Animal Care and Use Committee.

### Ambient whole-body inhalational protocol

Both C57BL/6 and CCR2-/- mice were exposed by inhalation to either filtered air (FA) or concentrated ambient PM_2.5_ for 6 h/d, 5 d/week from December 9, 2010 to June 8, 2011, for a total duration of 182 days, in a mobile trailer exposure system (“Ohio Air Pollution Exposure System for Interrogation of Systemic Effects 1,” located at the Ohio State University Animal Facility in Columbus). Chambers of the exposure system receives either concentrated PM_2.5_ directly from ambient air of Columbus site or filtered air. The exposure system is within the urban environment with relatively stable levels of ambient PM_2.5_ at about 10 μg/m^3^ [1, 3, 14]. The mean concentration of ambient black carbon was about 0.8 μg/m^3^ and the main elemental constituents were S, Ca, Na, Fe, K, Zn [28]. The animal groups are named as WT-FA (*n* = 10), WT-PM (*n* = 10), CCR2-FA (*n* = 8) and CCR2-PM (*n* = 6) for ease of identification throughout the manuscript. Animal exposure and monitoring of the exposure environment and ambient aerosol were performed as previously described [1, 3, 14].

#### Sampling and analysis of PM_2.5_ in the exposure chamber

To calculate PM_2.5_ mass concentrations in the exposure chambers, samples were collected weekly on Teflon filters (Teflon, 37 mm, 2 ɥm pore; Pall Life Sciences, Ann Arbor, MI, USA) and weighed before and after sampling in a temperature- and humidity-controlled weighing room using a microbalance (Mettle Toledo, Columbus, OH, USA). Samples collected from Teflon filters were wetted with ethanol and extracted in 1% nitric acid solution. The extraction solution was sonicated for 48 h in an ultrasonic bath and then allowed to passively acid digest for a minimum of 2 weeks. Sample extracts were then analyzed for a suite of trace elements using inductively coupled plasma-mass spectrometry (ELEMENT2, Thermo Finnigan, San Jose, CA, USA) as described [29].

### Measurements of blood glucose homeostasis and insulin sensitivity

Before, during and subsequent to the exposure to FA or PM_2.5_, mice were fasted overnight and dextrose (2 mg/g body weight) was injected intra-peritoneally for Intra-peritoneal Glucose Tolerance Test. Blood sample was collected from the vena caudalis and blood glucose measurement was conducted with an Contour Blood Glucose Meter (Bayer, Mishawaka, IN) at baseline, and 30, 60, 90, and 120 min after the dextrose injection. Insulin sensitivity was measured by the insulin-tolerance test. After 4.5 h fasting, human regular insulin (0.5 U/kg) (Novolin R, Clayton, NC) was administered by intra-peritoneal injection. Blood glucose measurement was conducted in the same way as Intra-peritoneal Glucose Tolerance Test. with the same Contour Blood Glucose Meter at baseline, and 30, 60, 90, and 120 min after insulin injection. At the end of experiment, liver and epididymal adipose tissue were harvested and immediately frozen in liquid nitrogen.

### Measurement of blood inflammatory biomarkers and adipokines

Circulating cytokine levels were determined by Cytometric Bead Array (BD Biosciences, San Diego, CA). Plasma was incubated with beads specific for tumor necrosis factor (TNFα), interferon γ (IFN-γ), IL-12p70, IL-10, IL-6 and MCP-1 according to the manufacturer’s instructions. The total amount of cytokines was then determined using a BD LSR II instrument and analyzed by the BD CBA software (BD Biosciences). Leptin and adiponectin levels in plasma were determined by the leptin quantification kit (Abcam, Cambridge, MA) or adiponectin quantification kit (R&D, Minneapolis, MN) following the manufacturer’s instructions.

### Immunoblotting

Protein levels were determined by western blot. Adipose tissue and liver were homogenized with M-PER Mammalian protein extraction reagent (Thermo Scientific) on ice. Equal quantities of protein for respective tissue were separated by 10% SDS-PAGE. Following transfer to immobilon-P polyvinylidene difluoride (PVDF) membrane and blocking with 5% nonfat milk, the blot was incubated with primary antibody: P-AKT, AKT, P-FoxO1 and FoxO1 (all antibodies at a concentration of 1:1000, from cell signaling). The immunoblots were then incubated with a secondary antibody conjugated with horseradish peroxidase and visualized with enhanced chemiluminescence, and the autoradiograph was quantitated by densitometric analysis with ImageJ software. β-actin was used as control reference.

### Quantitative RT-PCR

RT-PCR was performed using RNA extracted from adipose tissue of the experimental mice. Total RNA was isolated with Trizol (Invitrogen, Carlsbad, CA, USA) according to the manufacturer’s protocol. cDNA was reversely transcribed using High Capacity cDNA Transcription kit (Applied Biosystems, Carlsbad, California, USA). Quantitative polymerase chain reaction (qPCR) was performed in duplicate using the lightcycler 480. “No template,” cDNA negative controls were included for each gene set in all PCR reactions to detect contamination. The expression level for each gene was calculated using the ΔCt method relative to β-actin. The sequences of all primers used are listed in Table 1.

**Table 1:** Primers used for real-time PCR

Primer	Forward oligonucleotides	Reverse oligonucleotides
*PEPCK*	CCACAGCTGGTGCAGAACA	GAAGGGTCGATGGCAAA
*G6pase*	CCATGCAAAGGACTAGGAACAA	TACCAGGGCCGATGTCAAC
*FBPase*	AGGAAGCACAAAGCCAAGTGAAGG	TGAGGATGAAGTGACCTTGGGCAT
*PC*	GATGACCTCACAGCCAAGCA	GGGTACCTCTGTGTCCAAAGGA
*C/EBPα*	CAAGAACAGCAACGAGTACCG	TCACGGCTCAGCTGTTCCAC
*PGC1α*	GAGAATGAGGCAAACTTGCTAGCG	TGCATGGTTCTGAGTGCTAAGACC
*P67*	CAGTCCCAAGGAGAATGGAA	TCTGCCATAGCTGGACAGTG
*P47*	CTCAGCCAGGACACCTATCG	GGTGCAGGATGAGGTCTGAG
*P22*	GCTCATCTGTCTGCTGGAGTA	ACGACCTCATCTGTCACTGGC
*Gp91*	TGCCAACTTCCTCAGCTACA	TTGCAATGGTCTTGAACTCG
*Nox1*	CACGAGTGGGATGACCATAAG	CCCTGCTGCTCGAATATGAA
*Nox4*	TTGGAGTTTTCTGCTGTGGA	TGGCATAGCACAGCTGTTTG
*β-actin*	TGTGATGGTGGGAATGGGTCAGAA	TGTGGTGCCAGATCTTCTCCATGT

### Monocytes subset assessment by flow cytometry

VAT from the mice were excised, minced, and digested with collagenase type II, and the stromal vascular fractions cells were isolated as described previously [30]. Bone marrow derived cells were collected by flushing the femur and tibia with PBS. These cells were centrifuged at 500 x g for 5 min. Whole blood was centrifuged at 500 x g, 4 °C for 5 min and plasma was collected. The remaining blood cells and the resulting pellet of stromal vascular fraction cells were re-suspended in 1X red blood cell lysis buffer (Biolegend, San Diego, CA), at room temperature for 3 min followed by addition of 1 X PBS and centrifugation. Then, blood cells and bone marrow cells were stained with anti-CD11b, anti-7/4 and anti-Ly6G, stromal vascular fraction cells were stained with anti-CD11C, CD206 (MR) and F4/80, both followed by incubation at room temperature for 45 min. Cells were subsequently washed with 1 X PBS and re-suspended in 1% neutral buffered formalin and run by flow cytometry (BD FACS LSR II™ flow cytometer, Becton Dickinson, San Jose, CA). Data was analyzed using BD FACS Diva software (Becton Dickinson, San Jose,CA). All antibodies were purchased from Biolegend, Miltenyi Biotec, or BD Bioscience.

### nCounter™ gene expression assay

nCounter GX Mouse Inflammation Kit from NanoString Technologies (Seattle, WA) was applied to test a comprehensive set of 179 inflammation-related genes in this study. The mouse probe sequences were screened against mouse RefSeq to eliminate potentially cross-hybridizing probes. Total RNA (100 ng) was hybridized to nCounter™ capture and reporter probes at 65 °C for 16 h. The hybridized products were purified and processed using an automated sample prep station, and the images were prepared using the NanoString Digital Analyzer according to the company’s standard gene expression assay protocol [http://www.nanostring.com]. The 179 mouse inflammation-related genes and 6 internal reference genes are detailed in Additional file 1: Table S2.

### nCounter™ data analysis

The counts were first normalized to six spiked-in positive controls to correct for experimental variability. A reference normalization factor was determined by first calculating the geometric mean of the positive controls for each sample, and then computing the arithmetic mean across all samples. The gene count for each sample was then normalized by dividing by the ratio of the geometric mean of the positive controls for the sample to the reference normalization factor. To account for the variability in RNA content, the normalized gene counts were further normalized against four endogenous control genes. This was performed by calculating the geometric mean of the endogenous controls for each sample, averaging across all samples, and generating an endogenous normalization factor by computing the ratio of the geometric mean of the endogenous controls to the average value as described above. Each target gene count was divided by this endogenous normalization factor to compute the final normalized target gene count reflective of the transcript level. The detailed gene expression analysis guidelines can be found on the NanoString Technologies website [http://www.nanostring.com].

### Data analysis

Data are means ± standard error of the mean for the number indicated. For most of the analysis, two-way ANOVA followed by stratified analysis was used with PM_2.5_ exposure, CCR2 deletion, and the interaction as the independent variables. For the analysis of PM_2.5_ exposure on IPGTT and ITT, an repeated measures analysis was additionally performed using all observations in the two-way ANOVA analysis: PM_2.5_ exposure, CCR2 deletion, PM_2.5_ x CCR2 deletion, and time. When the interaction is considered significant, a stratified analysis was performed at each time point of measurement. *t* test was used for the exact *P* values measurement when comparing WT-PM with WT-FA. Graphpad Prism software (Version 5) was used for the analyses. *P* value of <0.05 was considered statistically significant.

## Additional file

Additional file 1: Table S1. Elemental composition of PM_2.5_ in OASIS, Columbus, OH. Table S2. 179 inflammation-related genes and 6 internal reference genes for nanoString. (DOCX 32 kb)

## Abbreviations

Bcl6: B-cell leukemia/lymphoma 6
Ccl17: Chemokine (C-C motif) ligand 17
Ccl7: Chemokine (C-C motif) ligand 7
CCR2: CC-chemokine recptor 2
Cxcr4: Chemokine (C-X-C motif) receptor 4
Elk1: Member of ETS oncogene family
FA: Filtered air
HFD: High fat diet
IL-18: Interleukin 18
Il1r1: Interleukin 1 receptor, type I
Il1rn: Interleukin 1 receptor antagonist
Il6ra: Interleukin 6 receptor, alpha
IR: Insulin resistance
Maff: v-maf musculoaponeurotic fibrosarcoma oncogene family, protein F (avian)
Mafg: v-maf musculoaponeurotic fibrosarcoma oncogene family, protein G (avian) transcript variant 3
Masp2: Mannan-binding lectin serine peptidase 2
MCP: Monocyte chemoattractant protein
Mef2a: Myocyte enhancer factor 2A, transcript variant 4
MAPK: Mitogen-activated protein kinase
ND: Normal diet
Nfe2l2: Erythroid derived 2, like 2
PEPCK: Phosphoenolpyruvate carboxykinase
PM_2.5_: Fine particulate matter
VAT: Visceral adipose tissue
