# Supplementary material for: Particulate Air pollution mediated effects on insulin resistance in mice are independent of CCR2
Source: Part Fibre Toxicol. 2017 Mar 3;14:6. doi: 10.1186/s12989-017-0187-3 (PMC5335830; doi:10.1186/s12989-017-0187-3)
Supplement: Additional file 1: Table S1. — Elemental composition of PM2.5 in OASIS, Columbus, OH. Table S2. 179 inflammation-related genes and 6 internal reference genes for nanoString. (DOCX 32 kb) [file 12989_2017_187_MOESM1_ESM.docx]

Additional file 1: Table S1. Elemental composition of PM_2.5_ in OASIS, Columbus, OH

| Element | Mean | SD |
| --- | --- | --- |
| S | 8691.25 | 4999.72 |
| Ca | 832.12 | 478.35 |
| Fe | 627.46 | 281.8 |
| Na | 491.25 | 390.78 |
| Al | 405.23 | 207.38 |
| K | 339.32 | 150.89 |
| Mg | 250.18 | 145.86 |
| P | 157.86 | 80.84 |
| Zn | 108.41 | 52.47 |
| Mn | 27.99 | 14.22 |
| Pb | 24.46 | 13.13 |
| Cu | 21.87 | 10.85 |
| Cd | 16.84 | 19.7 |
| Ti | 16.63 | 12.17 |
| Cr | 9.86 | 3.02 |
| Se | 9.26 | 9.42 |
| As | 6.88 | 7.91 |
| Co | 6.41 | 6.64 |
| Sr | 4.53 | 2.83 |
| Ni | 3 | 3 |
| Ga | 0.56 | 4.13 |

PM_2.5_ was collected weekly. Units are ng/mg.

Additional file 1: Table S2. 179 inflammation-related genes and 6 internal reference genes for nanoString

| ***Official Symbol*** | **Accession** | **Official Full Name** |
| --- | --- | --- |
| *Atf2* | [NM_001025093.1](http://www.ncbi.nlm.nih.gov/entrez/viewer.fcgi?db=nucleotide&val=NM_001025093.1) | activating transcription factor 2 (Atf2), transcript variant 1 |
| *Bcl6* | [NM_009744.3](http://www.ncbi.nlm.nih.gov/entrez/viewer.fcgi?db=nucleotide&val=NM_009744.3) | B-cell leukemia/lymphoma 6 (Bcl6) |
| *C1qa* | [NM_007572.2](http://www.ncbi.nlm.nih.gov/entrez/viewer.fcgi?db=nucleotide&val=NM_007572.2) | complement component 1, q subcomponent, alpha polypeptide (C1qa) |
| *C1qb* | [NM_009777.2](http://www.ncbi.nlm.nih.gov/entrez/viewer.fcgi?db=nucleotide&val=NM_009777.2) | complement component 1, q subcomponent, beta polypeptide (C1qb) |
| *C1r* | [NM_023143.3](http://www.ncbi.nlm.nih.gov/entrez/viewer.fcgi?db=nucleotide&val=NM_023143.3) | complement component 1, r subcomponent (C1r) |
| *C1s* | [NM_144938.2](http://www.ncbi.nlm.nih.gov/entrez/viewer.fcgi?db=nucleotide&val=NM_144938.2) | complement component 1, s subcomponent (C1s), transcript variant 1 |
| *C2* | [NM_013484.2](http://www.ncbi.nlm.nih.gov/entrez/viewer.fcgi?db=nucleotide&val=NM_013484.2) | complement component 2 (within H-2S) (C2) |
| *C3* | [NM_009778.2](http://www.ncbi.nlm.nih.gov/entrez/viewer.fcgi?db=nucleotide&val=NM_009778.2) | complement component 3 (C3) |
| *C3ar1* | [NM_009779.2](http://www.ncbi.nlm.nih.gov/entrez/viewer.fcgi?db=nucleotide&val=NM_009779.2) | complement component 3a receptor 1 (C3ar1) |
| *C4a* | [NM_011413.2](http://www.ncbi.nlm.nih.gov/entrez/viewer.fcgi?db=nucleotide&val=NM_011413.2) | complement component 4A (Rodgers blood group) (C4a) |
| *C6* | [NM_016704.2](http://www.ncbi.nlm.nih.gov/entrez/viewer.fcgi?db=nucleotide&val=NM_016704.2) | complement component 6 (C6) |
| *C7* | [XM_356827.6](http://www.ncbi.nlm.nih.gov/entrez/viewer.fcgi?db=nucleotide&val=XM_356827.6) | PREDICTED: complement component 7 (C7) |
| *C8a* | [NM_146148.1](http://www.ncbi.nlm.nih.gov/entrez/viewer.fcgi?db=nucleotide&val=NM_146148.1) | complement component 8, alpha polypeptide (C8a) |
| *C8b* | [NM_133882.2](http://www.ncbi.nlm.nih.gov/entrez/viewer.fcgi?db=nucleotide&val=NM_133882.2) | complement component 8, beta polypeptide (C8b) |
| *C9* | [NM_013485.1](http://www.ncbi.nlm.nih.gov/entrez/viewer.fcgi?db=nucleotide&val=NM_013485.1) | complement component 9 (C9) |
| *Ccl11* | [NM_011330.3](http://www.ncbi.nlm.nih.gov/entrez/viewer.fcgi?db=nucleotide&val=NM_011330.3) | chemokine (C-C motif) ligand 11 (Ccl11) |
| *Ccl17* | [NM_011332.2](http://www.ncbi.nlm.nih.gov/entrez/viewer.fcgi?db=nucleotide&val=NM_011332.2) | chemokine (C-C motif) ligand 17 (Ccl17) |
| *Ccl19* | [NM_011888.2](http://www.ncbi.nlm.nih.gov/entrez/viewer.fcgi?db=nucleotide&val=NM_011888.2) | chemokine (C-C motif) ligand 19 (Ccl19) |
| *Ccl2* | [NM_011333.3](http://www.ncbi.nlm.nih.gov/entrez/viewer.fcgi?db=nucleotide&val=NM_011333.3) | chemokine (C-C motif) ligand 2 (Ccl2) |
| *Ccl21b* | [NM_011124.4](http://www.ncbi.nlm.nih.gov/entrez/viewer.fcgi?db=nucleotide&val=NM_011124.4) | chemokine (C-C motif) ligand 21b (Ccl21b) |
| *Ccl22* | [NM_009137.2](http://www.ncbi.nlm.nih.gov/entrez/viewer.fcgi?db=nucleotide&val=NM_009137.2) | chemokine (C-C motif) ligand 22 (Ccl22) |
| *Ccl24* | [NM_019577.4](http://www.ncbi.nlm.nih.gov/entrez/viewer.fcgi?db=nucleotide&val=NM_019577.4) | chemokine (C-C motif) ligand 24 (Ccl24) |
| *Ccl3* | [NM_011337.1](http://www.ncbi.nlm.nih.gov/entrez/viewer.fcgi?db=nucleotide&val=NM_011337.1) | chemokine (C-C motif) ligand 3 (Ccl3) |
| *Ccl4* | [NM_013652.1](http://www.ncbi.nlm.nih.gov/entrez/viewer.fcgi?db=nucleotide&val=NM_013652.1) | chemokine (C-C motif) ligand 4 (Ccl4) |
| *Ccl5* | [NM_013653.1](http://www.ncbi.nlm.nih.gov/entrez/viewer.fcgi?db=nucleotide&val=NM_013653.1) | chemokine (C-C motif) ligand 5 (Ccl5) |
| *Ccl7* | [NM_013654.2](http://www.ncbi.nlm.nih.gov/entrez/viewer.fcgi?db=nucleotide&val=NM_013654.2) | chemokine (C-C motif) ligand 7 (Ccl7) |
| *Ccl8* | [NM_021443.2](http://www.ncbi.nlm.nih.gov/entrez/viewer.fcgi?db=nucleotide&val=NM_021443.2) | chemokine (C-C motif) ligand 8 (Ccl8) |
| *Ccr1* | [NM_009912.4](http://www.ncbi.nlm.nih.gov/entrez/viewer.fcgi?db=nucleotide&val=NM_009912.4) | chemokine (C-C motif) receptor 1 (Ccr1) |
| *Ccr2* | [NM_009915.2](http://www.ncbi.nlm.nih.gov/entrez/viewer.fcgi?db=nucleotide&val=NM_009915.2) | chemokine (C-C motif) receptor 2 (Ccr2) |
| *Ccr3* | [NM_009914.4](http://www.ncbi.nlm.nih.gov/entrez/viewer.fcgi?db=nucleotide&val=NM_009914.4) | chemokine (C-C motif) receptor 3 (Ccr3) |
| *Ccr4* | [NM_009916.2](http://www.ncbi.nlm.nih.gov/entrez/viewer.fcgi?db=nucleotide&val=NM_009916.2) | chemokine (C-C motif) receptor 4 (Ccr4) |
| *Ccr7* | [NM_007719.2](http://www.ncbi.nlm.nih.gov/entrez/viewer.fcgi?db=nucleotide&val=NM_007719.2) | chemokine (C-C motif) receptor 7 (Ccr7) |
| *Cd4* | [NM_013488.2](http://www.ncbi.nlm.nih.gov/entrez/viewer.fcgi?db=nucleotide&val=NM_013488.2) | CD4 antigen (Cd4) |
| *Cd40* | [NM_011611.2](http://www.ncbi.nlm.nih.gov/entrez/viewer.fcgi?db=nucleotide&val=NM_011611.2) | CD40 antigen (Cd40), transcript variant 1 |
| *Cd40lg* | [NM_011616.2](http://www.ncbi.nlm.nih.gov/entrez/viewer.fcgi?db=nucleotide&val=NM_011616.2) | CD40 ligand (Cd40lg) |
| *Cd55* | [NM_010016.2](http://www.ncbi.nlm.nih.gov/entrez/viewer.fcgi?db=nucleotide&val=NM_010016.2) | CD55 antigen (Cd55) |
| *Cdc42* | [NM_009861.1](http://www.ncbi.nlm.nih.gov/entrez/viewer.fcgi?db=nucleotide&val=NM_009861.1) | cell division cycle 42 homolog (S. cerevisiae) (Cdc42) |
| *Cebpb* | [NM_009883.3](http://www.ncbi.nlm.nih.gov/entrez/viewer.fcgi?db=nucleotide&val=NM_009883.3) | CCAAT/enhancer binding protein (C/EBP), beta (Cebpb) |
| *Cfb* | [NM_008198.2](http://www.ncbi.nlm.nih.gov/entrez/viewer.fcgi?db=nucleotide&val=NM_008198.2) | complement factor B (Cfb), transcript variant 1 |
| *Cfd* | [NM_013459.1](http://www.ncbi.nlm.nih.gov/entrez/viewer.fcgi?db=nucleotide&val=NM_013459.1) | complement factor D (adipsin) (Cfd) |
| *Cfl1* | [NM_007687.2](http://www.ncbi.nlm.nih.gov/entrez/viewer.fcgi?db=nucleotide&val=NM_007687.2) | cofilin 1, non-muscle (Cfl1) |
| *Creb1* | [NM_133828.2](http://www.ncbi.nlm.nih.gov/entrez/viewer.fcgi?db=nucleotide&val=NM_133828.2) | cAMP responsive element binding protein 1 (Creb1), transcript variant A |
| *Crp* | [NM_007768.4](http://www.ncbi.nlm.nih.gov/entrez/viewer.fcgi?db=nucleotide&val=NM_007768.4) | C-reactive protein, pentraxin-related (Crp) |
| *Csf1* | [NM_001113530.1](http://www.ncbi.nlm.nih.gov/entrez/viewer.fcgi?db=nucleotide&val=NM_001113530.1) | colony stimulating factor 1 (macrophage) (Csf1), transcript variant 3 |
| *Csf2* | [NM_009969.4](http://www.ncbi.nlm.nih.gov/entrez/viewer.fcgi?db=nucleotide&val=NM_009969.4) | colony stimulating factor 2 (granulocyte-macrophage) (Csf2) |
| *Csf3* | [NM_009971.1](http://www.ncbi.nlm.nih.gov/entrez/viewer.fcgi?db=nucleotide&val=NM_009971.1) | colony stimulating factor 3 (granulocyte) (Csf3) |
| *Cxcl1* | [NM_008176.1](http://www.ncbi.nlm.nih.gov/entrez/viewer.fcgi?db=nucleotide&val=NM_008176.1) | chemokine (C-X-C motif) ligand 1 (Cxcl1) |
| *Cxcl10* | [NM_021274.1](http://www.ncbi.nlm.nih.gov/entrez/viewer.fcgi?db=nucleotide&val=NM_021274.1) | chemokine (C-X-C motif) ligand 10 (Cxcl10) |
| *Cxcl2* | [NM_009140.2](http://www.ncbi.nlm.nih.gov/entrez/viewer.fcgi?db=nucleotide&val=NM_009140.2) | chemokine (C-X-C motif) ligand 2 (Cxcl2) |
| *Cxcl3* | [NM_203320.2](http://www.ncbi.nlm.nih.gov/entrez/viewer.fcgi?db=nucleotide&val=NM_203320.2) | chemokine (C-X-C motif) ligand 3 (Cxcl3) |
| *Cxcl5* | [NM_009141.2](http://www.ncbi.nlm.nih.gov/entrez/viewer.fcgi?db=nucleotide&val=NM_009141.2) | chemokine (C-X-C motif) ligand 5 (Cxcl5) |
| *Cxcl9* | [NM_008599.2](http://www.ncbi.nlm.nih.gov/entrez/viewer.fcgi?db=nucleotide&val=NM_008599.2) | chemokine (C-X-C motif) ligand 9 (Cxcl9) |
| *Cxcr4* | [NM_009911.3](http://www.ncbi.nlm.nih.gov/entrez/viewer.fcgi?db=nucleotide&val=NM_009911.3) | chemokine (C-X-C motif) receptor 4 (Cxcr4) |
| *Daxx* | [NM_007829.3](http://www.ncbi.nlm.nih.gov/entrez/viewer.fcgi?db=nucleotide&val=NM_007829.3) | Fas death domain-associated protein (Daxx) |
| *Ddit3* | [NM_007837.3](http://www.ncbi.nlm.nih.gov/entrez/viewer.fcgi?db=nucleotide&val=NM_007837.3) | DNA-damage inducible transcript 3 (Ddit3) |
| *Elk1* | [NM_007922.4](http://www.ncbi.nlm.nih.gov/entrez/viewer.fcgi?db=nucleotide&val=NM_007922.4) | ELK1, member of ETS oncogene family (Elk1) |
| *Fasl* | [NM_010177.3](http://www.ncbi.nlm.nih.gov/entrez/viewer.fcgi?db=nucleotide&val=NM_010177.3) | Fas ligand (TNF superfamily, member 6) (Fasl) |
| *Fos* | [NM_010234.2](http://www.ncbi.nlm.nih.gov/entrez/viewer.fcgi?db=nucleotide&val=NM_010234.2) | FBJ osteosarcoma oncogene (Fos) |
| *Fxyd2* | [NM_052823.2](http://www.ncbi.nlm.nih.gov/entrez/viewer.fcgi?db=nucleotide&val=NM_052823.2) | FXYD domain-containing ion transport regulator 2 (Fxyd2), transcript variant b |
| *Gnaq* | [NM_008139.5](http://www.ncbi.nlm.nih.gov/entrez/viewer.fcgi?db=nucleotide&val=NM_008139.5) | guanine nucleotide binding protein, alpha q polypeptide (Gnaq) |
| *Gnas* | [NM_010309.3](http://www.ncbi.nlm.nih.gov/entrez/viewer.fcgi?db=nucleotide&val=NM_010309.3) | GNAS (guanine nucleotide binding protein, alpha stimulating) complex locus (Gnas), transcript variant 1 |
| *Gnb1* | [NM_008142.3](http://www.ncbi.nlm.nih.gov/entrez/viewer.fcgi?db=nucleotide&val=NM_008142.3) | guanine nucleotide binding protein, beta 1 (Gnb1) |
| *Gngt1* | [NM_010314.2](http://www.ncbi.nlm.nih.gov/entrez/viewer.fcgi?db=nucleotide&val=NM_010314.2) | guanine nucleotide binding protein (G protein), gamma transducing activity polypeptide 1 (Gngt1) |
| *Grb2* | [NM_008163.3](http://www.ncbi.nlm.nih.gov/entrez/viewer.fcgi?db=nucleotide&val=NM_008163.3) | growth factor receptor bound protein 2 (Grb2) |
| *H2-Ea* | [NM_010381.2](http://www.ncbi.nlm.nih.gov/entrez/viewer.fcgi?db=nucleotide&val=NM_010381.2) | histocompatibility 2, class II antigen E alpha (H2-Ea) |
| *H2-Eb1* | [NM_010382.2](http://www.ncbi.nlm.nih.gov/entrez/viewer.fcgi?db=nucleotide&val=NM_010382.2) | histocompatibility 2, class II antigen E beta (H2-Eb1) |
| *Hc* | [NM_010406.1](http://www.ncbi.nlm.nih.gov/entrez/viewer.fcgi?db=nucleotide&val=NM_010406.1) | hemolytic complement (Hc) |
| *Hdac4* | [NM_207225.1](http://www.ncbi.nlm.nih.gov/entrez/viewer.fcgi?db=nucleotide&val=NM_207225.1) | histone deacetylase 4 (Hdac4) |
| *Hmgn1* | [NM_008251.3](http://www.ncbi.nlm.nih.gov/entrez/viewer.fcgi?db=nucleotide&val=NM_008251.3) | high mobility group nucleosomal binding domain 1 (Hmgn1) |
| *Hras1* | [NM_008284.2](http://www.ncbi.nlm.nih.gov/entrez/viewer.fcgi?db=nucleotide&val=NM_008284.2) | Harvey rat sarcoma virus oncogene 1 (Hras1), transcript variant 1 |
| *Hspb1* | [NM_013560.2](http://www.ncbi.nlm.nih.gov/entrez/viewer.fcgi?db=nucleotide&val=NM_013560.2) | heat shock protein 1 (Hspb1) |
| *Hspb2* | [NM_024441.2](http://www.ncbi.nlm.nih.gov/entrez/viewer.fcgi?db=nucleotide&val=NM_024441.2) | heat shock protein 2 (Hspb2) |
| *Ifna1* | [NM_010502.2](http://www.ncbi.nlm.nih.gov/entrez/viewer.fcgi?db=nucleotide&val=NM_010502.2) | interferon alpha 1 (Ifna1) |
| *Ifnb1* | [NM_010510.1](http://www.ncbi.nlm.nih.gov/entrez/viewer.fcgi?db=nucleotide&val=NM_010510.1) | interferon beta 1, fibroblast (Ifnb1) |
| *Ifng* | [NM_008337.1](http://www.ncbi.nlm.nih.gov/entrez/viewer.fcgi?db=nucleotide&val=NM_008337.1) | interferon gamma (Ifng) |
| *Il10* | [NM_010548.1](http://www.ncbi.nlm.nih.gov/entrez/viewer.fcgi?db=nucleotide&val=NM_010548.1) | interleukin 10 (Il10) |
| *Il10rb* | [NM_008349.5](http://www.ncbi.nlm.nih.gov/entrez/viewer.fcgi?db=nucleotide&val=NM_008349.5) | interleukin 10 receptor, beta (Il10rb) |
| *Il11* | [NM_008350.2](http://www.ncbi.nlm.nih.gov/entrez/viewer.fcgi?db=nucleotide&val=NM_008350.2) | interleukin 11 (Il11) |
| *Il12a* | [NM_008351.1](http://www.ncbi.nlm.nih.gov/entrez/viewer.fcgi?db=nucleotide&val=NM_008351.1) | interleukin 12a (Il12a) |
| *Il12b* | [NM_008352.1](http://www.ncbi.nlm.nih.gov/entrez/viewer.fcgi?db=nucleotide&val=NM_008352.1) | interleukin 12b (Il12b) |
| *Il13* | [NM_008355.2](http://www.ncbi.nlm.nih.gov/entrez/viewer.fcgi?db=nucleotide&val=NM_008355.2) | interleukin 13 (Il13) |
| *Il15* | [NM_008357.1](http://www.ncbi.nlm.nih.gov/entrez/viewer.fcgi?db=nucleotide&val=NM_008357.1) | interleukin 15 (Il15) |
| *Il18* | [NM_008360.1](http://www.ncbi.nlm.nih.gov/entrez/viewer.fcgi?db=nucleotide&val=NM_008360.1) | interleukin 18 (Il18) |
| *Il18rap* | [NM_010553.2](http://www.ncbi.nlm.nih.gov/entrez/viewer.fcgi?db=nucleotide&val=NM_010553.2) | interleukin 18 receptor accessory protein (Il18rap) |
| *Il1a* | [NM_010554.4](http://www.ncbi.nlm.nih.gov/entrez/viewer.fcgi?db=nucleotide&val=NM_010554.4) | interleukin 1 alpha (Il1a) |
| *Il1b* | [NM_008361.3](http://www.ncbi.nlm.nih.gov/entrez/viewer.fcgi?db=nucleotide&val=NM_008361.3) | interleukin 1 beta (Il1b) |
| *Il1r1* | [NM_001123382.1](http://www.ncbi.nlm.nih.gov/entrez/viewer.fcgi?db=nucleotide&val=NM_001123382.1) | interleukin 1 receptor, type I (Il1r1), transcript variant 2 |
| *Il1rap* | [NM_008364.2](http://www.ncbi.nlm.nih.gov/entrez/viewer.fcgi?db=nucleotide&val=NM_008364.2) | interleukin 1 receptor accessory protein (Il1rap), transcript variant 1 |
| *Il1rn* | [NM_031167.4](http://www.ncbi.nlm.nih.gov/entrez/viewer.fcgi?db=nucleotide&val=NM_031167.4) | interleukin 1 receptor antagonist (Il1rn), transcript variant 1 |
| *Il2* | [NM_008366.2](http://www.ncbi.nlm.nih.gov/entrez/viewer.fcgi?db=nucleotide&val=NM_008366.2) | interleukin 2 (Il2) |
| *Il22* | [NM_016971.1](http://www.ncbi.nlm.nih.gov/entrez/viewer.fcgi?db=nucleotide&val=NM_016971.1) | interleukin 22 (Il22) |
| *Il22ra2* | [NM_178258.5](http://www.ncbi.nlm.nih.gov/entrez/viewer.fcgi?db=nucleotide&val=NM_178258.5) | interleukin 22 receptor, alpha 2 (Il22ra2) |
| *Il23a* | [NM_031252.1](http://www.ncbi.nlm.nih.gov/entrez/viewer.fcgi?db=nucleotide&val=NM_031252.1) | interleukin 23, alpha subunit p19 (Il23a) |
| *Il23r* | [NM_144548.1](http://www.ncbi.nlm.nih.gov/entrez/viewer.fcgi?db=nucleotide&val=NM_144548.1) | interleukin 23 receptor (Il23r) |
| *Il3* | [NM_010556.4](http://www.ncbi.nlm.nih.gov/entrez/viewer.fcgi?db=nucleotide&val=NM_010556.4) | interleukin 3 (Il3) |
| *Il4* | [NM_021283.1](http://www.ncbi.nlm.nih.gov/entrez/viewer.fcgi?db=nucleotide&val=NM_021283.1) | interleukin 4 (Il4) |
| *Il5* | [NM_010558.1](http://www.ncbi.nlm.nih.gov/entrez/viewer.fcgi?db=nucleotide&val=NM_010558.1) | interleukin 5 (Il5) |
| *Il6* | [NM_031168.1](http://www.ncbi.nlm.nih.gov/entrez/viewer.fcgi?db=nucleotide&val=NM_031168.1) | interleukin 6 (Il6) |
| *Il6ra* | [NM_010559.2](http://www.ncbi.nlm.nih.gov/entrez/viewer.fcgi?db=nucleotide&val=NM_010559.2) | interleukin 6 receptor, alpha (Il6ra) |
| *Il7* | [NM_008371.2](http://www.ncbi.nlm.nih.gov/entrez/viewer.fcgi?db=nucleotide&val=NM_008371.2) | interleukin 7 (Il7) |
| *Il8ra* | [NM_178241.4](http://www.ncbi.nlm.nih.gov/entrez/viewer.fcgi?db=nucleotide&val=NM_178241.4) | interleukin 8 receptor, alpha (Il8ra) |
| *Il8rb* | [NM_009909.3](http://www.ncbi.nlm.nih.gov/entrez/viewer.fcgi?db=nucleotide&val=NM_009909.3) | interleukin 8 receptor, beta (Il8rb) |
| *Il9* | [NM_008373.1](http://www.ncbi.nlm.nih.gov/entrez/viewer.fcgi?db=nucleotide&val=NM_008373.1) | interleukin 9 (Il9) |
| *Itgb2* | [NM_008404.4](http://www.ncbi.nlm.nih.gov/entrez/viewer.fcgi?db=nucleotide&val=NM_008404.4) | integrin beta 2 (Itgb2) |
| *Jun* | [NM_010591.2](http://www.ncbi.nlm.nih.gov/entrez/viewer.fcgi?db=nucleotide&val=NM_010591.2) | Jun oncogene (Jun) |
| *Keap1* | [NM_016679.4](http://www.ncbi.nlm.nih.gov/entrez/viewer.fcgi?db=nucleotide&val=NM_016679.4) | kelch-like ECH-associated protein 1 (Keap1), transcript variant 1 |
| *Kng1* | [NM_023125.3](http://www.ncbi.nlm.nih.gov/entrez/viewer.fcgi?db=nucleotide&val=NM_023125.3) | kininogen 1 (Kng1), transcript variant 2 |
| *Limk1* | [NM_010717.2](http://www.ncbi.nlm.nih.gov/entrez/viewer.fcgi?db=nucleotide&val=NM_010717.2) | LIM-domain containing, protein kinase (Limk1) |
| *Lta* | [NM_010735.1](http://www.ncbi.nlm.nih.gov/entrez/viewer.fcgi?db=nucleotide&val=NM_010735.1) | lymphotoxin A (Lta) |
| *Ltb* | [NM_008518.2](http://www.ncbi.nlm.nih.gov/entrez/viewer.fcgi?db=nucleotide&val=NM_008518.2) | lymphotoxin B (Ltb) |
| *Ly96* | [NM_016923.1](http://www.ncbi.nlm.nih.gov/entrez/viewer.fcgi?db=nucleotide&val=NM_016923.1) | lymphocyte antigen 96 (Ly96) |
| *Maff* | [NM_010755.3](http://www.ncbi.nlm.nih.gov/entrez/viewer.fcgi?db=nucleotide&val=NM_010755.3) | v-mafmusculoaponeuroticfibrosarcoma oncogene family, protein F (avian) (Maff) |
| *Mafg* | [XM_001002362.1](http://www.ncbi.nlm.nih.gov/entrez/viewer.fcgi?db=nucleotide&val=XM_001002362.1) | PREDICTED: v-mafmusculoaponeuroticfibrosarcoma oncogene family, protein G (avian), transcript variant 3 (Mafg) |
| *Mafk* | [NM_010757.2](http://www.ncbi.nlm.nih.gov/entrez/viewer.fcgi?db=nucleotide&val=NM_010757.2) | v-mafmusculoaponeuroticfibrosarcoma oncogene family, protein K (avian) (Mafk) |
| *Map2k1* | [NM_008927.3](http://www.ncbi.nlm.nih.gov/entrez/viewer.fcgi?db=nucleotide&val=NM_008927.3) | mitogen-activated protein kinase kinase 1 (Map2k1) |
| *Map2k4* | [NM_009157.4](http://www.ncbi.nlm.nih.gov/entrez/viewer.fcgi?db=nucleotide&val=NM_009157.4) | mitogen-activated protein kinase kinase 4 (Map2k4) |
| *Map2k6* | [NM_011943.2](http://www.ncbi.nlm.nih.gov/entrez/viewer.fcgi?db=nucleotide&val=NM_011943.2) | mitogen-activated protein kinase kinase 6 (Map2k6) |
| *Map3k1* | [NM_011945.2](http://www.ncbi.nlm.nih.gov/entrez/viewer.fcgi?db=nucleotide&val=NM_011945.2) | mitogen activated protein kinase kinasekinase 1 (Map3k1) |
| *Map3k5* | [NM_008580.4](http://www.ncbi.nlm.nih.gov/entrez/viewer.fcgi?db=nucleotide&val=NM_008580.4) | mitogen-activated protein kinase kinasekinase 5 (Map3k5) |
| *Map3k7* | [NM_172688.2](http://www.ncbi.nlm.nih.gov/entrez/viewer.fcgi?db=nucleotide&val=NM_172688.2) | mitogen-activated protein kinase kinasekinase 7 (Map3k7) |
| *Map3k9* | [NM_177395.4](http://www.ncbi.nlm.nih.gov/entrez/viewer.fcgi?db=nucleotide&val=NM_177395.4) | mitogen-activated protein kinase kinasekinase 9 (Map3k9) |
| *Mapk1* | [NM_001038663.1](http://www.ncbi.nlm.nih.gov/entrez/viewer.fcgi?db=nucleotide&val=NM_001038663.1) | mitogen activated protein kinase 1 (Mapk1), transcript variant 2 |
| *Mapk14* | [NM_011951.2](http://www.ncbi.nlm.nih.gov/entrez/viewer.fcgi?db=nucleotide&val=NM_011951.2) | mitogen-activated protein kinase 14 (Mapk14) |
| *Mapk3* | [NM_011952.2](http://www.ncbi.nlm.nih.gov/entrez/viewer.fcgi?db=nucleotide&val=NM_011952.2) | mitogen activated protein kinase 3 (Mapk3) |
| *Mapk8* | [NM_016700.3](http://www.ncbi.nlm.nih.gov/entrez/viewer.fcgi?db=nucleotide&val=NM_016700.3) | mitogen-activated protein kinase 8 (Mapk8) |
| *Mapkapk2* | [NM_008551.1](http://www.ncbi.nlm.nih.gov/entrez/viewer.fcgi?db=nucleotide&val=NM_008551.1) | MAP kinase-activated protein kinase 2 (Mapkapk2) |
| *Mapkapk5* | [XM_990515.1](http://www.ncbi.nlm.nih.gov/entrez/viewer.fcgi?db=nucleotide&val=XM_990515.1) | PREDICTED: MAP kinase-activated protein kinase 5 (Mapkapk5) |
| *Masp1* | [NM_008555.2](http://www.ncbi.nlm.nih.gov/entrez/viewer.fcgi?db=nucleotide&val=NM_008555.2) | mannan-binding lectin serine peptidase 1 (Masp1) |
| *Masp2* | [NM_010767.3](http://www.ncbi.nlm.nih.gov/entrez/viewer.fcgi?db=nucleotide&val=NM_010767.3) | mannan-binding lectin serine peptidase 2 (Masp2), transcript variant 2 |
| *Max* | [NM_008558.1](http://www.ncbi.nlm.nih.gov/entrez/viewer.fcgi?db=nucleotide&val=NM_008558.1) | Max protein (Max) |
| *Mbl2* | [NM_010776.1](http://www.ncbi.nlm.nih.gov/entrez/viewer.fcgi?db=nucleotide&val=NM_010776.1) | mannose binding lectin (C) (Mbl2) |
| *Mef2a* | [XM_976032.1](http://www.ncbi.nlm.nih.gov/entrez/viewer.fcgi?db=nucleotide&val=XM_976032.1) | PREDICTED: myocyte enhancer factor 2A, transcript variant 4 (Mef2a) |
| *Mef2b* | [NM_001045484.1](http://www.ncbi.nlm.nih.gov/entrez/viewer.fcgi?db=nucleotide&val=NM_001045484.1) | myocyte enhancer factor 2B (Mef2b), transcript variant 2 |
| *Mef2c* | [NM_025282.2](http://www.ncbi.nlm.nih.gov/entrez/viewer.fcgi?db=nucleotide&val=NM_025282.2) | myocyte enhancer factor 2C (Mef2c) |
| *Mef2d* | [NM_133665.3](http://www.ncbi.nlm.nih.gov/entrez/viewer.fcgi?db=nucleotide&val=NM_133665.3) | myocyte enhancer factor 2D (Mef2d) |
| *Mknk1* | [NM_021461.4](http://www.ncbi.nlm.nih.gov/entrez/viewer.fcgi?db=nucleotide&val=NM_021461.4) | MAP kinase-interacting serine/threonine kinase 1 (Mknk1) |
| *Myc* | [NM_010849.4](http://www.ncbi.nlm.nih.gov/entrez/viewer.fcgi?db=nucleotide&val=NM_010849.4) | myelocytomatosis oncogene (Myc) |
| *Myd88* | [NM_010851.2](http://www.ncbi.nlm.nih.gov/entrez/viewer.fcgi?db=nucleotide&val=NM_010851.2) | myeloid differentiation primary response gene 88 (Myd88) |
| *Myl2* | [NM_010861.3](http://www.ncbi.nlm.nih.gov/entrez/viewer.fcgi?db=nucleotide&val=NM_010861.3) | myosin, light polypeptide 2, regulatory, cardiac, slow (Myl2) |
| *Nfatc3* | [NM_010901.2](http://www.ncbi.nlm.nih.gov/entrez/viewer.fcgi?db=nucleotide&val=NM_010901.2) | nuclear factor of activated T-cells, cytoplasmic, calcineurin-dependent 3 (Nfatc3) |
| *Nfe2l2* | [NM_010902.3](http://www.ncbi.nlm.nih.gov/entrez/viewer.fcgi?db=nucleotide&val=NM_010902.3) | nuclear factor, erythroid derived 2, like 2 (Nfe2l2) |
| *Nfkb1* | [NM_008689.2](http://www.ncbi.nlm.nih.gov/entrez/viewer.fcgi?db=nucleotide&val=NM_008689.2) | nuclear factor of kappa light chain gene enhancer in B-cells 1, p105 (Nfkb1) |
| *Nos2* | [NM_010927.3](http://www.ncbi.nlm.nih.gov/entrez/viewer.fcgi?db=nucleotide&val=NM_010927.3) | nitric oxide synthase 2, inducible (Nos2) |
| *Nox1* | [NM_172203.1](http://www.ncbi.nlm.nih.gov/entrez/viewer.fcgi?db=nucleotide&val=NM_172203.1) | NADPH oxidase 1 (Nox1) |
| *Nr3c1* | [NM_008173.3](http://www.ncbi.nlm.nih.gov/entrez/viewer.fcgi?db=nucleotide&val=NM_008173.3) | nuclear receptor subfamily 3, group C, member 1 (Nr3c1) |
| *Pdgfa* | [NM_008808.3](http://www.ncbi.nlm.nih.gov/entrez/viewer.fcgi?db=nucleotide&val=NM_008808.3) | platelet derived growth factor, alpha (Pdgfa) |
| *Pik3c2g* | [NM_011084.2](http://www.ncbi.nlm.nih.gov/entrez/viewer.fcgi?db=nucleotide&val=NM_011084.2) | phosphatidylinositol 3-kinase, C2 domain containing, gamma polypeptide (Pik3c2g), transcript variant 2 |
| *Pla2g4a* | [NM_008869.2](http://www.ncbi.nlm.nih.gov/entrez/viewer.fcgi?db=nucleotide&val=NM_008869.2) | phospholipase A2, group IVA (cytosolic, calcium-dependent) (Pla2g4a) |
| *Plcb1* | [NM_019677.1](http://www.ncbi.nlm.nih.gov/entrez/viewer.fcgi?db=nucleotide&val=NM_019677.1) | phospholipase C, beta 1 (Plcb1) |
| *Ppp1r12b* | [NM_001081307.1](http://www.ncbi.nlm.nih.gov/entrez/viewer.fcgi?db=nucleotide&val=NM_001081307.1) | protein phosphatase 1, regulatory (inhibitor) subunit 12B (Ppp1r12b) |
| *Prkca* | [NM_011101.3](http://www.ncbi.nlm.nih.gov/entrez/viewer.fcgi?db=nucleotide&val=NM_011101.3) | protein kinase C, alpha (Prkca) |
| *Prkcb1* | [NM_008855.2](http://www.ncbi.nlm.nih.gov/entrez/viewer.fcgi?db=nucleotide&val=NM_008855.2) | protein kinase C, beta 1 (Prkcb1) |
| *Ptk2* | [NM_007982.2](http://www.ncbi.nlm.nih.gov/entrez/viewer.fcgi?db=nucleotide&val=NM_007982.2) | PTK2 protein tyrosine kinase 2 (Ptk2), transcript variant 1 |
| *Rac1* | [NM_009007.2](http://www.ncbi.nlm.nih.gov/entrez/viewer.fcgi?db=nucleotide&val=NM_009007.2) | RAS-related C3 botulinum substrate 1 (Rac1) |
| *Raf1* | [NM_029780.3](http://www.ncbi.nlm.nih.gov/entrez/viewer.fcgi?db=nucleotide&val=NM_029780.3) | v-raf-leukemia viral oncogene 1 (Raf1) |
| *Rapgef2* | [NM_001099624.2](http://www.ncbi.nlm.nih.gov/entrez/viewer.fcgi?db=nucleotide&val=NM_001099624.2) | Rap guanine nucleotide exchange factor (GEF) 2 (Rapgef2) |
| *Rhoa* | [NM_016802.4](http://www.ncbi.nlm.nih.gov/entrez/viewer.fcgi?db=nucleotide&val=NM_016802.4) | ras homolog gene family, member A (Rhoa) |
| *Ripk1* | [NM_009068.3](http://www.ncbi.nlm.nih.gov/entrez/viewer.fcgi?db=nucleotide&val=NM_009068.3) | receptor (TNFRSF)-interacting serine-threonine kinase 1 (Ripk1) |
| *Ripk2* | [NM_138952.3](http://www.ncbi.nlm.nih.gov/entrez/viewer.fcgi?db=nucleotide&val=NM_138952.3) | receptor (TNFRSF)-interacting serine-threonine kinase 2 (Ripk2) |
| *Rock2* | [NM_009072.2](http://www.ncbi.nlm.nih.gov/entrez/viewer.fcgi?db=nucleotide&val=NM_009072.2) | Rho-associated coiled-coil containing protein kinase 2 (Rock2) |
| *Rps6ka5* | [NM_153587.2](http://www.ncbi.nlm.nih.gov/entrez/viewer.fcgi?db=nucleotide&val=NM_153587.2) | ribosomal protein S6 kinase, polypeptide 5 (Rps6ka5) |
| *Shc1* | [NM_011368.4](http://www.ncbi.nlm.nih.gov/entrez/viewer.fcgi?db=nucleotide&val=NM_011368.4) | src homology 2 domain-containing transforming protein C1 (Shc1), transcript variant 2 |
| *Stat1* | [NM_009283.3](http://www.ncbi.nlm.nih.gov/entrez/viewer.fcgi?db=nucleotide&val=NM_009283.3) | signal transducer and activator of transcription 1 (Stat1) |
| *Tgfb1* | [NM_011577.1](http://www.ncbi.nlm.nih.gov/entrez/viewer.fcgi?db=nucleotide&val=NM_011577.1) | transforming growth factor, beta 1 (Tgfb1) |
| *Tgfb2* | [NM_009367.1](http://www.ncbi.nlm.nih.gov/entrez/viewer.fcgi?db=nucleotide&val=NM_009367.1) | transforming growth factor, beta 2 (Tgfb2) |
| *Tgfb3* | [NM_009368.2](http://www.ncbi.nlm.nih.gov/entrez/viewer.fcgi?db=nucleotide&val=NM_009368.2) | transforming growth factor, beta 3 (Tgfb3) |
| *Tgfbr1* | [NM_009370.2](http://www.ncbi.nlm.nih.gov/entrez/viewer.fcgi?db=nucleotide&val=NM_009370.2) | transforming growth factor, beta receptor I (Tgfbr1) |
| *Tlr1* | [NM_030682.1](http://www.ncbi.nlm.nih.gov/entrez/viewer.fcgi?db=nucleotide&val=NM_030682.1) | toll-like receptor 1 (Tlr1) |
| *Tlr2* | [NM_011905.2](http://www.ncbi.nlm.nih.gov/entrez/viewer.fcgi?db=nucleotide&val=NM_011905.2) | toll-like receptor 2 (Tlr2) |
| *Tlr3* | [NM_126166.2](http://www.ncbi.nlm.nih.gov/entrez/viewer.fcgi?db=nucleotide&val=NM_126166.2) | toll-like receptor 3 (Tlr3) |
| *Tlr4* | [NM_021297.2](http://www.ncbi.nlm.nih.gov/entrez/viewer.fcgi?db=nucleotide&val=NM_021297.2) | toll-like receptor 4 (Tlr4) |
| *Tlr5* | [NM_016928.2](http://www.ncbi.nlm.nih.gov/entrez/viewer.fcgi?db=nucleotide&val=NM_016928.2) | toll-like receptor 5 (Tlr5) |
| *Tlr6* | [NM_011604.3](http://www.ncbi.nlm.nih.gov/entrez/viewer.fcgi?db=nucleotide&val=NM_011604.3) | toll-like receptor 6 (Tlr6) |
| *Tlr7* | [NM_133211.3](http://www.ncbi.nlm.nih.gov/entrez/viewer.fcgi?db=nucleotide&val=NM_133211.3) | toll-like receptor 7 (Tlr7) |
| *Tnf* | [NM_013693.1](http://www.ncbi.nlm.nih.gov/entrez/viewer.fcgi?db=nucleotide&val=NM_013693.1) | tumor necrosis factor (Tnf) |
| *Tnfsf14* | [NM_019418.2](http://www.ncbi.nlm.nih.gov/entrez/viewer.fcgi?db=nucleotide&val=NM_019418.2) | tumor necrosis factor (ligand) superfamily, member 14 (Tnfsf14) |
| *Tollip* | [NM_023764.3](http://www.ncbi.nlm.nih.gov/entrez/viewer.fcgi?db=nucleotide&val=NM_023764.3) | toll interacting protein (Tollip) |
| *Tradd* | [NM_001033161.2](http://www.ncbi.nlm.nih.gov/entrez/viewer.fcgi?db=nucleotide&val=NM_001033161.2) | TNFRSF1A-associated via death domain (Tradd) |
| *Traf2* | [NM_009422.2](http://www.ncbi.nlm.nih.gov/entrez/viewer.fcgi?db=nucleotide&val=NM_009422.2) | Tnf receptor-associated factor 2 (Traf2) |
|  |  |  |
| *Internal Reference Genes* | |  |
| *Cltc* | [NM_001003908.1](http://www.ncbi.nlm.nih.gov/entrez/viewer.fcgi?db=nucleotide&val=NM_001003908.1) | clathrin, heavy polypeptide (Hc) |
| *Gapdh* | [NM_008084.1](http://www.ncbi.nlm.nih.gov/entrez/viewer.fcgi?db=nucleotide&val=NM_008084.1) | glyceraldehyde-3-phosphate dehydrogenase |
| *Gusb* | [NM_010368.1](http://www.ncbi.nlm.nih.gov/entrez/viewer.fcgi?db=nucleotide&val=NM_010368.1) | glucuronidase, beta |
| *Hprt1* | [NM_013556.2](http://www.ncbi.nlm.nih.gov/entrez/viewer.fcgi?db=nucleotide&val=NM_013556.2) | hypoxanthine guanine phosphoribosyltransferase |
| *Pgk1* | [NM_008828.2](http://www.ncbi.nlm.nih.gov/entrez/viewer.fcgi?db=nucleotide&val=NM_008828.2) | phosphoglycerate kinase 1 |
| *Tubb5* | [NM_011655.4](http://www.ncbi.nlm.nih.gov/entrez/viewer.fcgi?db=nucleotide&val=NM_011655.4) | tubulin, beta 5 |
